# Evaluating the influence of static management on individuals’ oral health

**DOI:** 10.1186/s12903-023-03300-8

**Published:** 2023-08-23

**Authors:** Tu Huang, Juan Li, Zihao Wang

**Affiliations:** 1 Department of Stomatology, Chengdu Women’s and Children’s Central Hospital, School of Medicine, University of Electronic Science and Technology of China, Chengdu, 611731 China; 2 Department of Stomatology, Southwest Medical University, 363 Hospital, Chengdu, China; 3 Chengdu Jinjiang center for disease control and prevention, Chengdu, China

**Keywords:** COVID-19 pandemics, Depression, Oral health, Quality of life, Government Regulation, Social Support

## Abstract

**Purpose:**

This study aimed to evaluate the effect of static management on individuals’ oral health-related quality of life (OHRQoL) according to the dynamic zero-COVID policy in China.

**Methods:**

The digital questionnaire conducted with three sub-questionnaires was sent to 700 patients who accepted treatment at the Department of Stomatology, 363 Hospital. Data on demographic characteristics, the Oral Health Impact Profile-14 and willingness to invest in oral health were collected from the 658 completed questionnaires. According to the state of individuals’ lives, participants were divided into two groups: a static management group (Group 1) and a nonstatic management group (Group 2). The scores of the Oral Health Impact Profile-14 and willingness to invest in oral health were compared between these two groups using IBM SPSS Statistics.

**Results:**

The results showed that individuals undergoing static management reported better OHRQoL. Meanwhile, they also presented lower willingness to invest money and dental visits in oral health. Furthermore, according to the results of the logistic regression analysis, aging acts as a negative correlation factor for the OHRQoL of people undergoing static management, while the willingness to invest money and dental visits in oral health is defined as a positive predictor for OHRQoL.

**Conclusion:**

Static management effects the OHRQoL of individuals. Aging and WTIOH in money and dental visits are related the individuals’ OHRQoL during static management.

## Introduction

Coronavirus disease 2019 (COVID-19) has been defined as a respiratory disease caused by the virus SARS-CoV-2 since its outbreak [1]. The global burden of COVID-19 cases caused by the COVID-19 pandemic has been an issue of concern for more than 3 years [2].

Previous studies have revealed the influence of the COVID-19 pandemic on individuals’ oral health [1, 3]. First, a series of oral lesions, including herpex simplex, candidiasis, aphthous-like ulcers and reddish macules, have been reported to be related to COVID-19 infection [4–7]. Although there was not enough evidence showing that these manifestations were directly induced by viral infection, some patients with a history of COVID-19 were exposed to the discomfort and pain caused by these oral lesions. Second, the COVID-19 pandemic has impacted different areas all over the world, including globalization, economics, manufacturing, tourism and people’s private lives [2, 3, 8, 9]. In the early day of the COVID-19 pandemic, people in most countries experienced a prolonged lockdown period. At that time, people were asked to stay at home and stop most of the social contact [1]. In addition, business and public services had also been reduced to a minimum, including dental care [1]. Hence, it was difficult for individuals who suffered from caries, periodontal disease or other oral diseases to seek for medical care. With the development of prevention and therapy for COVID-19, contact restrictions have been cancelled in most countries, and people seem to return to their pre-pandemic lives. However, a study by Nikolić et al. showed that the majority of individuals defined dental offices as high-risk places and hotspots for the transmission of COVID-19. More than 50% of participants chose to avoid dental visits as long as possible. Thus, dental care avoidance caused by the fear of COVID-19 infection impacts the prevention and treatment of oral diseases, whether or not contact restrictions are in place. Furthermore, mental stress caused by the COVID-19 pandemic was another negative factor for individuals’ oral health. The study of Ciardo et al. showed that the COVID-19 pandemic brought different levels of depression, anxiety, and stress, leading to poorer OHRQoL compared to pre-pandemic levels [1]. Overall, both direct and indirect effects of COVID-19 were related to people’s oral health. Reviews focusing on the impact of COVID-19 on oral health have demonstrated that the indirect effects of COVID-19, such as the various government responses and the subsequent societal response, were likely to be of huge consequence for individuals’ oral health rather than its direct effects [7].

Different from most countries, China has adhered to the dynamic zero-COVID policy and adopted precise and differentiated epidemic control strategies since the epidemic outbreak of disease. According to the dynamic zero-COVID policy, some cities or districts in China may enter static management when local cases continue to surge. In static management, people are asked to stay at home and public services have also been reduced to a minimum, including dental care. Based on data from the World Health Organization, 609 million cases have been reported as of mid-September 2022. In particular, China was seeing new daily cases soaring to 1000 by the end of November 2022 from less than 100 before. The high morbidity rate and uncertainty of the mutated strains makes it highly likely that a large number of Chinese people would undergo static management for several weeks or even months [2]. According to the specialized dynamic zero-COVID policy, the majority of Chinese had not been infected with COVID-19 until the end of November 2022. Thus, this study provided a rare sample to study the indirect effect of COVID-19 on individuals’ oral health and their oral health-related quality of life (OHRQoL).

## Materials and methods

### Study design and sample

The present quantitative cross-sectional study was conducted in the city of Chengdu, southwestern China. The participants were selected randomly from these patients who had accepted dental care at the Department of Stomatology, 363 Hospital. The questionnaire was sent to 700 patients by E-mail and was online available for eight weeks from September 1, 2022, to November 1, 2022. A total of 658 questionnaires were completed by patients who volunteered to participate in the study. The inclusion criteria were as follows: (1) Those who were residents in enclosed or opening districts in China; (2) Volunteered to participate in research. Exclusion criteria included (1) Those who did not finish the questionnaire within the required time; (2) Those who suffered from other chronic painful disorders or cognitive impairment.

This study included two groups: a static management group (Group 1) and a nonstatic management group (Group 2). Group 1 was composed of 401 participants who had been in a static management state for at least 2 weeks. Group 2 consisted of 257 participants who had been in a normal state of personal life.

### Data collection

In this study, a digital questionnaire consisting of three sub-questionnaires was used for data collection. Information on demographic and socioeconomic characteristics was obtained by questions in the first part of the questionnaire, such as gender (female or male), age (in years), maternal education, and statement of life (static or nonstatic). A question related to income change was also assessed: “Has the income increased or decreased in the past six months?”, posteriorly categorized as increased or constant or decreased (reflected by decreased or increased financial stress).

In the second part, participants’ OHRQoL was evaluated by the Chinese version of the Oral Health Impact Profile-14 (OHIP-14) questionnaire. The OHIP-14 questionnaire has been widely used to assess the impact of oral health problems on an individual’s life [10]. Cronbach’s alpha of its Chinese version was 0.93, showing good reliability and validity [11]. This questionnaire evaluates seven dimensions of oral health impact, including functional limitation, physical pain, psychological discomfort, physical disability, psychological disability, social disability, and handicap, through 14 validated questions [12–14]. A 5-point Likert-like scale was used to collect answers from participants (0 stands for never occurred, 1 stands for hardly ever, 2 stands for occasionally, 3 stands for fairly often, and 4 stands for very often) [15, 16]. The total score of the OHIP-14 was calculated by adding the scores of all 14 items, ranging from 0 to 56. The domain scores were calculated by adding the scores of the two questions belonging to the same dimension, ranging from 0 to 8. Individuals with higher scores were suggested to suffer poorer OHRQoL.

In the third part, the willingness to invest in oral health (WTIOH) of the participants was assessed by an existing set of questions [17, 18]. Two dimensions of participants’WTIOH, including willingness to pay and willingness to invest in time were measured by this set of questions. The self-administered questionnaire consists three closed-ended questions with predefined response options.

1. “How much are you willing to pay to keep your oral health every month?

Answer options: ¥0; ¥1–¥20; ¥21–¥50; ¥51–¥100; or more than ¥100.

2. “How many times are you willing to visit dentist for check-ups or treatment to keep your oral health every month?

Answer options: 0visit; 1visit; 2visits; 4visits; or more than 4 visits.

3. “How many minutes are you willing to brush your teeth to keep your oral health every day?”

Answer options: 0 min; 1–2 min; 3–4 min; or more than 4 min a day.

The midpoint of the payment and time was used for the following statistical analysis (e.g., RMB 1–20 has been recoded to RMB 10.5, RMB 21–50 has been recoded to RMB 35.5, RMB 51–100 has been recoded to RMB 75.5 and more than RMB 100 to RMB 101) [18].

### Statistical analysis

IBM SPSS Statistics (v25.0 for Windows; IBM Corp) was used for statistical analysis in this study. First, frequencies (percentages) of demographic data were evaluated. The Pearson chi-square and Fisher exact tests were used to compare the baseline characteristics of the two groups. In the second and third part, the comparison of the OHIP-14 scores and WTIOH was conducted by the nonparametric Mann‒Whitney U test. In addition, the strength of the association between OHRQoL and sociodemographic factors and the strength of the correlation between OHRQoL and WTIOH was tested by binary logistic regression analysis. Median splits were used to dichotomize the OHIP-14 scores. Lower OHIP-14 scores were recorded as 0, and higher OHIP-14 scores were recorded as 1 for the following logistic regression analysis. Predictors of OHIP-14 from sociodemographic and WTIOH factors were shown in the logistic regression analysis, and the calculated odds ratios (ORs) and 95% confidence intervals (95% CI) of these factors were presented in the results.

## Results

The results of demographic data in both groups were presented in Table 1. No significant difference was found in sex, age, level of education, or change in income between the two groups (P > 0.05).

**Table 1:** Demographic data

Variables	G1 (n = 401)	G2 (n = 257)	P
	N (%)	N (%)	
Gender	—	—	0.576
Male	186 (46.4%)	107 (41.6%)	—
Female	215 (53.6%)	150 (58.4%)	—
Age (years)	—	—	0.089
18–22	55 (13.7%)	43 (16.7%)	—
23–45	239 (59.6%)	164 (63.8%)	—
>45	107 (26.7%)	50 (19.5%)	—
Education	—	—	0.776
Bachelor degree or below	374 (93.3%)	237 (59.1%)	—
Master	23 (5.7%)	18 (4.5%)	—
Doctor degree	4 (1.0%)	2 (0.5%)	—
Income	—	—	0.066
Increased	5 (1.2%)	4 (1.6%)	—
Constant	286 (71.3%)	161 (62.6%)	—
Decreased	110 (27.4%)	92 (35.8%)	—

Bold indicates statistical significance at P < 0.05 for Pearson chi-square or Fisher exact test. *p < 0.05 **p < 0.01

The results of OHIP-14 scores, including the total scores and domain scores of OHIP-14, were shown in Table 2. The mean total OHIP-14 score of the 658 participants was 3.87 ± 8.09. Depending on the state of individuals’ life, significant differences were detected for total OHIP-14 scores (Group 1/Group 2 3.43/4.56; p < 0.001) and four of the seven domains, including functional limitation (P=<0.001), physical pain (P = 0.011), psychological discomfort (P=<0.001), and physical disability (P = 0.009), between the two groups. The results of OHIP-14 scores revealed the phenomenon that individuals undergoing static management reported better OHRQoL, particularly in the domains of functional limitation, physical pain, psychological discomfort, and physical disability, when compared with people who enjoyed normal life.

**Table 2:** Comparison of OHIP-14 scores between the two groups

OHIP-14 Domain	G1 (n = 401)	G2 (n = 257)	P
OHIP-14 total score	0 (0–2)	0 (0–6)	<0.001**
Functional limitation	0 (0–0)	0 (0–1)	0.011*
Physical pain	0 (0–0)	0 (0–2)	<0.001**
Psychological discomfort	0 (0–0)	0(0–1)	0.009**
physical disability	0 (0–0)	0 (0–1)	0.005**
Psychological disability	0 (0–0)	0 (0–0)	0.057
Social disability	0 (0–0)	0 (0–0)	0.489
Handicap	0 (0–0)	0 (0–0)	0.133

The median and 25th to 75th percentile. **Bold** indicates statistical significance at P < 0.05 for the Mann‒Whitney U test. *p < 0.05 **p < 0.01

The results of the WTIOH questions were shown in Tables 3 and 4. In terms of tooth brushing, the majority of the participants were willing to invest at least 1–2 min per day to maintain their oral health. However, nearly half of the participants reported that they did not want to invest any time or money in oral health. The results of the Mann‒Whitney U test were shown in Table 4. Depending on the state of individuals’ lives, significantly higher WTIOH in money and dental visits per month were reported in group 2 than in group 1.

**Table 3:** WTIOH data

Willingness to invest	N (%)	Interval Midpoint WTP and WTIT	
Willingness to pay			
RMB 0 per month	209 (31.76)	Mean (SD)	39.65 (39.94)
RMB 1–20 per month	113 (17.17)	95% CI	36.59–42.71
RMB 20–50 per month	88 (13.37)	Median (IQ)	35 (75)
RMB 50–100 per month	124 (18.84)		
More than RMB100 per month	124 (18.84)		
Willingness to visit the dentist			
0 visit per month	388 (59.0)	Mean (SD)	0.66 (1.09)
1 visit per month	185 (28.1)	95% CI	0.58–0.75
2 visits per month	55 (8.4)	Median (IQ)	0 (1)
4 visits per month	9 (1.4)		
More than 4 visits per month	21 (3.2)		
Willingness to brush			
0 min per day	85 (12.9)	Mean (SD)	3.18 (1.74)
1–2 min per day	127 (19.3)	95% CI	3.05–3.32
3–4 min per day	217 (33.0)	Median (IQ)	3.5 (3.5)
More than 4 min per day	229 (34.8)		

**Table 4:** Comparison of WTIOH between the two groups

Willingness to invest in oral health	G1 (n = 401)	G2 (n = 257)	P
RMB per month	10.5 (0–75)	35 (0–75)	0.002**
Visit per month	0 (0–1)	1 (0–1)	0.002**
minute every day	3.5(1.5-5)	3.5 (1.5-5)	0.197

The median and 25th to 75th percentile. **Bold** indicates statistical significance at P < 0.05 for the Mann‒Whitney U test. *p < 0.05 **p < 0.01

The results of the binary logistic regression analysis revealed that age, WTIOH in money and dental visits per month were significantly associated with OHRQoL for individuals in group 1 who underwent static management caused by the COVID-19 pandemic (details in Table 5). According to the binary logistic regression analysis, higher WTIOH in money per month was associated with better OHRQoL (1 to 20 RMB per month: OR: 0.308, 95% CI: 0.144–0.659, P = 0.002; 20 to 50 RMB per month: OR: 0.323, 95% CI: 0.143–0.730, P = 0.007), while aging (23–45 years old: OR: 2.884, 95% CI: 1.371–6.066, P = 0.005) and higher WTIOH (2 visits per month: OR: 4.281, 95% CI: 1.083–16.927, P = 0.038) in dental visits per month were associated with significantly poorer OHRQoL.

**Table 5:** Predictors associated with logistic regression analysis

Variables	Overall		
	OR	95% CI for OR	*P*
Age (in years)	——	——	——
18–22	reference	——	——
23–45	2.884	1.371–6.066	0.005**
>45	1.45	0.818–2.57	0.203
Willingness to pay	——	——	——
RMB 0 per month	reference	——	——
RMB 1–20 per month	0.308	0.144–0.659	0.002**
RMB 20–50 per month	0.323	0.143–0.73	0.007**
RMB 50–100 per month	0.578	0.255–1.307	0.188
More than RMB100 per month	0.665	0.313–1.415	0.29
Willingness to visit the dentist	——	——	——
0 visit per month	reference	——	——
1 visit per month	2.198	0.572–8.451	0.252
2 visits per month	4.281	1.083–16.927	0.038*
4 visits per month	3.713	0.864–15.947	0.078
More than 4 visits per month	0.507	0.043–6.031	0.591

CI, confidence interval; OR, odds ratio. Bold indicates statistical significance at P < 0.05. *p < 0.05 **p < 0.01

## Discussion

This study explored the association of COVID-19 pandemic caused static management and OHRQoL along with exploring the predictors of OHRQoL in this association. The present study reported that OHIP-14 total scores showed significant differences between the static management and nonstatic management groups, reflecting the different levels of OHRQoL among these two groups. Significant differences were also found among 4 domain scores of the OHIP-14, including functional limitation, physical pain, psychological discomfort, and physical disability.

In China, public and private lives have been partly restricted by lockdown and contact restrictions since the beginning of the COVID-19 pandemic [1]. In particular, the number of new cases in China surged to 1000 a day by the end of November 2022 from less than 100 previously [19]. Hence, a large number of residents had been undergoing static management since September 2022 according to the dynamic zero-COVID policy. To our knowledge, the associations of static management and OHRQoL under differentiated epidemic control strategies in China were first investigated in this study.

The OHIP-14 was defined as a reliable, sensitive, and accurate questionnaire that has been widely used in numbers of studies, which were focusing on OHRQoL [13]. The OHRQoL of individuals was evaluated by the Chinese version of the OHIP-14. Analysis of the OHIP-14 data revealed a mean sum score of 3.87 in this study. Since the 4.37 was identified as the mean sum score of the OHIP-14 for young adults in China, participants in this study reported a higher level of OHRQoL [20]. The results of the Mann‒Whitney U test presented significant differences in the OHIP-14 total scores and domain scores between the two groups, indicating that individuals undergoing static management reported better OHRQoL than participants in group 2. Although the results of this study were different from those of several previous studies, a study in Brazil reported a similar result [1]. Knorst et al. announced that the COVID-19 pandemic had reduced the negative perception of OHRQoL in adolescents from southern Brazil [21].

**Fig. 1:**
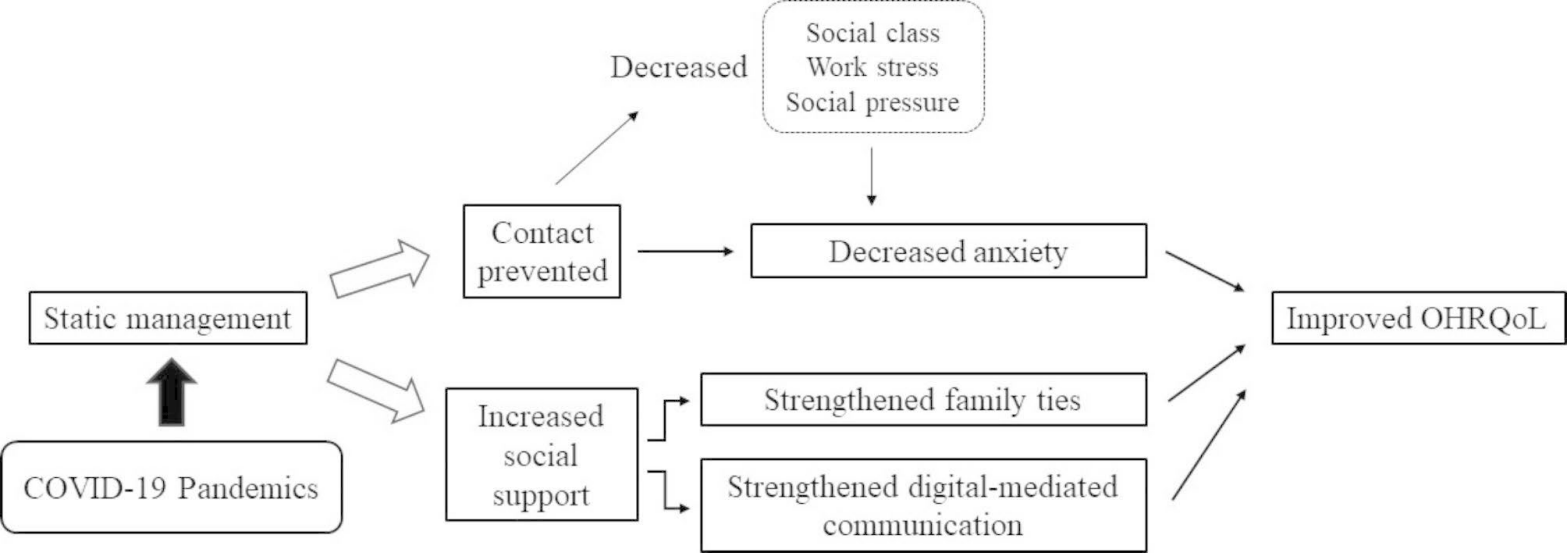
Diagram illustrating the correlation between COVID-19, static management, and OHRQoL.

Several theories can be used to explain this finding (Fig. 1). First, OHRQoL is a multidimensional tool evaluating the impact of oral health on individuals’ daily lives, including functional well-being, emotional well-being, and sense of self [21]. As individuals stopped most of the production activities and social contact during static management, a number of factors that may affect OHRQoL in regular life have changed during static management [21]. For example, Knorst et al. announced that social anxiety disorder was a negative factor for psychological health and led to poor OHRQoL [21]. In this sense, as daily contact among individuals was prevented by static management, it was believed that the impact of social anxiety disorder on OHRQoL was limited [21]. In spite of social anxiety disorder, many factors have been defined to be related to individuals’ OHRQoL, including social class, psychosocial wellbeing and work stress [21–24]. All of these potential factors, which may negatively affect individuals’ OHRQoL, were limited during static management.

Another theory of social capital also provides an explanation for this finding. Previous studies have shown that networks of strong ties could benefit individuals when facing accidents, including natural disasters and pandemics [25, 26]. Since individuals were asked to be home during static management, it was highly likely that they strengthened their family ties and receive increased social support [21, 25–27]. Depending on strengthened social networks, individuals were able to deal with stress caused by the COVID-19 pandemic and report better OHRQoL. In addition, digital-mediated communication played an important role in generating social capital and offers social support for people when facing static management [25]. Individuals may spend more time on digital-mediated communication tools since the majority of them were asked to work or rest at home. Therefore, they could strengthen their remote connection with friends, classmates and colleagues from schools or workplaces.

Another finding of this study was that people in the nonstatic group showed a higher willingness to pay money and dental visits for oral health than people in the static group. Several possible explanations can be explored for this result of WTIOH. First, poorer OHRQoL is related to a higher willingness to invest in oral health. Oscarson et al. announced that patients with caries experience reported higher values for mean yearly willingness to pay than patients with healthy teeth [28]. In this context, as individuals in group 2 reported poorer OHRQoL, it was reasonable to hypothesize that they were suffering from some kind of oral diseases and seeking treatment. Therefore, people with regular life showed higher WTIOH than participants undergoing static management. Another explanation is that the static management caused by the COVID-19 pandemic intensified the dental anxiety of potential patients. Investigation focusing on the attitude of patients toward dental visits during the COVID-19 pandemic showed that the majority of individuals defined dental offices as high-risk places and hotspots for the transmission of COVID-19 [29]. Thus, nearly 50% of patients presented fear of dental visits during the pandemic, which was significantly higher than that in the days before the outbreak [29]. In addition, static management was found to be a risk factor for individuals’ financial crises [29, 30]. Thus, it is natural that people would rather put their money on necessities, for example, food supplies and accommodations, than seeking dental treatment.

Aging was an essential factor impacting individuals’ OHRQoL during static management. Several studies have reported the same results. For example, the studies of Tabesh et al. and Xu et al. have reported that OHRQoL deteriorates as patients age [31, 32]. Two potential mechanisms can be used to explain the association between aging and OHRQoL. First, younger individuals were able to obtain more scientific knowledge of oral hygiene and health care than older individuals with the development of society. Thus, it was easier for them to prevent and control oral diseases at the early stage, which could keep their oral health and improve individuals’ OHRQoL [33]. On the other hand, since older individuals always suffered from tooth loss, xerostomia, and even systematic diseases, they may be bothered by pain and eating disabilities for a long time. Therefore, there was a high rate that they would report poorer OHRQoL caused by oral or systematic diseases [33].

WTIOH in money and dental visits served as a positive predictor in the association of static management and OHRQoL. Individuals who were willing to invest more money or dental visits reported better OHRQoL. There are two potential theories that can explain this result. First, we can assume that the WTIOH of individuals reflected their attitude toward oral health. Individuals with higher WTIOH in money and dental visits may pay more attention to their oral health. Thus, it was more likely for them to take measures to prevent and control oral diseases, including the purchase of oral hygiene products and healthcare for preventing oral diseases [18]. Another possible explanation is related to socioeconomic status and level of education [29]. According to the study of Vermaire et al., individuals with higher socioeconomic status and level of education were reported to have higher willingness to invest [17]. These people could obtain more knowledge of oral health and more opportunities to prevent and control oral diseases, attributed to their socioeconomic status. As a consequence, individuals with higher WTIOH in money and dental visits may report better OHRQoL benefiting from regular healthcare and promoted treatment.

The major strength of this study was that we focused on the special population and evaluated the indirect effects of COVID-19 on individuals’ oral health during static management. People who were not statically managed at the same time were set as the control group. To our knowledge, this was the first study evaluating the effects of citywide static management on individuals’ OHRQoL. Since China has released the control of the epidemic at the end of December 2022, there were no more opportunities to collect data on temporary static management. Another strength of the study was the novel nature of the data. People undergoing static management reported better OHRQoL in this study, which was different from most of the published literature.

The limitation of this study was that no clinical data were collected. However, according to the design of this study, it was extremely difficult to obtain clinical data during static management. Continued assessments should be conducted at different intervals of the COVID-19 pandemic in future work. Furthermore, it will be helpful to supplement the clinical data of participants.

## Conclusions

Based on the findings of this clinical study, we can conclude that individuals undergoing static management reported lower OHIP-14 total scores, indicating better OHRQoL. People with higher WTIOH in money and dental visits reported better OHRQoL during static management. However, higher WTIOH in money and dental visits was found in people with normal life.

## Data Availability

The dataset(s) supporting the conclusions of this article is(are) included within the article (and its additional file(s)).
